# Mast cells decrease efficacy of anti-angiogenic therapy by secreting matrix-degrading granzyme B

**DOI:** 10.1038/s41467-017-00327-8

**Published:** 2017-08-16

**Authors:** M. Wroblewski, R. Bauer, M. Cubas Córdova, F. Udonta, I. Ben-Batalla, K. Legler, C. Hauser, J. Egberts, M. Janning, J. Velthaus, C. Schulze, K. Pantel, C. Bokemeyer, S. Loges

**Affiliations:** 1 Department of Hematology and Oncology with Sections BMT and Pneumology, Hubertus Wald Tumorzentrum, University Comprehensive Cancer Center Hamburg, University Medical Center Hamburg-Eppendorf, Martinistraße 52, 20246 Hamburg, Germany; 2 Institute of Tumor Biology, Center of Experimental Medicine University Medical Center Hamburg-Eppendorf, Martinistraße 52, 20246 Hamburg, Germany; 3 Division of Molecular Oncology, Institute for Experimental Cancer Research, University Medical Center Schleswig-Holstein (UKSH), Campus Kiel, Arnold-Heller-Straße 3, 25105 Kiel, Germany; 4 Department of General, Visceral-, Thoracic-, Transplantation- and Pediatric Surgery, University Medical Center Schleswig-Holstein (UKSH), Campus Kiel, Arnold-Heller-Straße 3, 25105 Kiel, Germany; 5 Center for Molecular Neurobiology Hamburg, University Medical Center Hamburg-Eppendorf, Falkenried 94, 20251 Hamburg, Germany; 6 Department of Gynecology, University Medical Center Hamburg-Eppendorf, Martinistraße 52, 20246 Hamburg, Germany

## Abstract

Resistance towards VEGF-centered anti-angiogenic therapy still represents a substantial clinical challenge. We report here that mast cells alter the proliferative and organizational state of endothelial cells which reduces the efficacy of anti-angiogenic therapy. Consequently, absence of mast cells sensitizes tumor vessels for anti-angiogenic therapy in different tumor models. Mechanistically, anti-angiogenic therapy only initially reduces tumor vessel proliferation, however, this treatment effect was abrogated over time as a result of mast cell-mediated restimulation of angiogenesis. We show that mast cells secrete increased amounts of granzyme b upon therapy, which mobilizes pro-angiogenic laminin- and vitronectin-bound FGF-1 and GM-CSF from the tumor matrix. In addition, mast cells also diminish efficacy of anti-angiogenic therapy by secretion of FGF-2. These pro-angiogenic factors act beside the targeted VEGFA–VEGFR2-axis and reinduce endothelial cell proliferation and angiogenesis despite the presence of anti-angiogenic therapy. Importantly, inhibition of mast cell degranulation with cromolyn is able to improve efficacy of anti-angiogenic therapy. Thus, concomitant mast cell-targeting might lead to improved efficacy of anti-angiogenic therapy.

## Abstract (web summary)

Resistance towards VEGF-centered anti-angiogenic therapy is an important clinical challenge. Here, the authors show that mast cells mediate resistance to anti-angiogenetic inhibitors by altering the proliferative and organizational state of endothelial cells through mobilization of FGF-1 and GM-CSF from the tumor matrix and secretion of FGF-2.

## Introduction

Tumor angiogenesis represents an attractive therapeutic target because of its negligible role in physiologic conditions^1, 2^. Anti-angiogenic therapy (AAT) became an important treatment option resulting in approval of more than ten angiogenesis inhibitors for cancer treatment^3^.

Due to the central role of VEGFA in pathological angiogenesis^4, 5^, the majority of approved anti-angiogenic drugs are either targeting VEGF or its receptors. Although many cancer patients substantially benefit from AAT, resistance remains an important clinical challenge. Some cancers including pancreatic cancer and breast cancer do not or only minimally respond to AAT^6–8^. Other cancer types such as colorectal cancer or renal cancer are more sensitive to this treatment but a significant fraction of patients still do not respond upfront and in responding patients the therapeutic benefits are mostly not durable^9, 10^. In the recent neoadjuvant Phase 3 Gepar Quinto (G5) trial, patients with HER2-negative newly diagnosed localized breast cancer were treated with standard chemotherapy with or without the anti-VEGF antibody bevacizumab^11^. Although addition of bevacizumab significantly improved the pCR rate, progression-free survival, and overall survival were not increased after 3.8 years of follow-up^12^.

Possible reasons for lack or loss of efficacy of anti-angiogenic drugs include hypoxia-triggered upregulation of pro-angiogenic factors besides the VEGF axis and the recruitment of resistance-conferring cell populations, such as tumor-associated macrophages, myeloid-derived suppressor cells (MDSC), or cancer-associated fibroblasts^1, 13–15^. However, targeting those cells remains difficult due to lack of specific inhibitors that could be used for clinical trials. Consequently, not much progress has been made so far in improving efficacy of angiogenesis inhibitors in cancer patients.

Mast cells (MC) have been considered as innocent bystanders in tumor biology for a long time. However, some studies link MC to angiogenesis, because they accumulate in tumors before the onset of angiogenesis and reside in close proximity to blood vessels^16^. In other investigations, MC density correlates with microvessel density (MVD) and prognosis of cancer patients^17, 18^. Furthermore, in a model of pancreatic cancer, genetic ablation of MC reduced intratumoral MVD, which led to decreased cancer growth^19^.

Here, we uncover that MC directly and indirectly mediate decreased sensitivity of tumors towards AAT. This mechanism hampers the efficacy of AAT and its inhibition could open up therapeutic avenues to improve AAT.

## Results

### Mast cells impair efficacy of AAT in vivo

In a first step, we analyzed if the presence of MC influences efficacy of the anti-VEGFR2 antibody DC101. Therefore, we injected Panc02 cells into the flanks of C57BL/6J (WT) or MC-deficient Kit^W-sh^ (Wsh) mice, and treated the mice with a sub-maximal dose of DC101 (20 mg/kg).

**Fig. 1:**
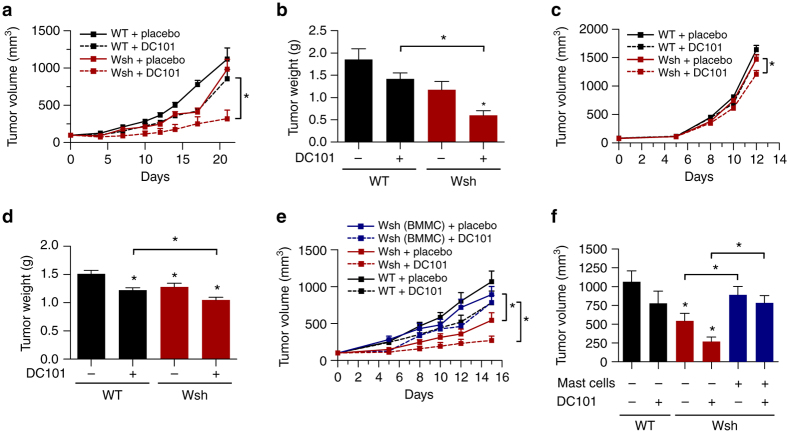
Mast cells impair the efficacy of AAT. **a** Subcutaneously growing Panc02 tumors. Cells were injected into the flanks of C57Bl/6J (WT) or MC-deficient (Wsh) mice and the animals received treatment with placebo or 20 mg/kg DC101 (*n* = 8/8/5/5; *p < 0.05; two-way ANOVA). **b** Weight of end-stage Panc02 tumors at day of euthanasia (*n* = 8/8/5/5; *p < 0.05; one-way ANOVA). **c** Subcutaneously growing EL4 tumors. Cells were injected into the flanks of C57Bl/6J (WT) or MC-deficient (Wsh) mice and the animals received treatment with placebo or 20 mg/kg DC101 (*n* = 5/5/5/5; *p < 0.05; two-way ANOVA). **d** Weight of end-stage EL4 tumors at day of euthanasia (*n* = 5/5/5/5; *p < 0.05; one-way ANOVA). **e**, **f** Kinetic (**e**) or end-stage (**f**) tumor volume of subcutaneously growing Panc02 tumors with or without adoptive transfer of MC. After randomization animals received treatment with placebo or 20 mg/kg DC101 (*n* = 9/9/7/6/7/5; *p < 0.05; two-way ANOVA and one-way ANOVA). Results are shown as representative means ± s.e.m

MC-deficiency reduced tumor growth by 37 ± 19% (*n* = 5–8; *p* = 0.07; one-way ANOVA). Interestingly, DC101 treatment reduced tumor volume and weight more pronouncedly in the absence of mast cells (WT: 24 ± 17%, *n* = 8, *p* = 0.19 and Wsh: 68 ± 12%, *n* = 5; *p < 0.05; one-way ANOVA) (Fig. 1a, b). The effect could be validated in a second syngeneic tumor model (EL4 lymphoma), although it was less pronounced (Fig. 1c, d).

By co-injecting fully functional bone marrow-derived MC (BMMC) (Supplementary Fig. 1a, b) and Panc02 cells into MC-deficient Wsh mice, we could restore tumor growth and decrease sensitivity of tumors towards DC101 to similar levels as observed in WT animals (Fig. 1e, f). Hence, MC specifically decrease sensitivity of tumors towards AAT.

### Mast cell-deficiency and AAT induce additive effects

In order to investigate the underlying reasons for the reduced efficacy of AAT in the presence of MC we performed histological analyzes of tumor sections.

**Fig. 2:**
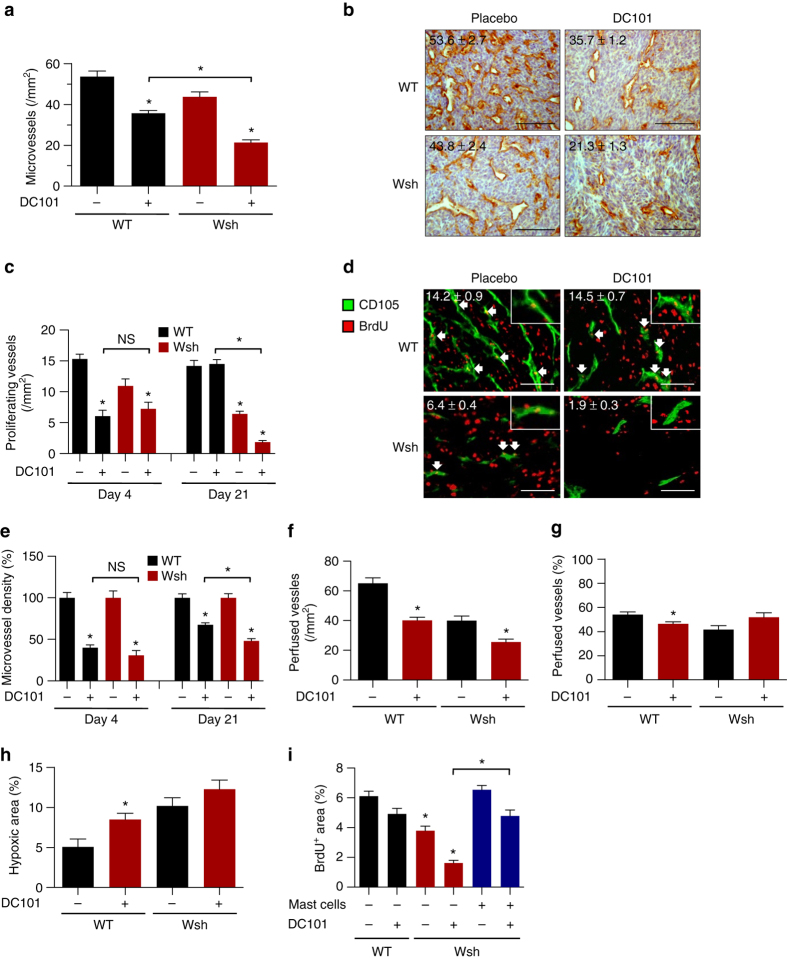
Mast cell-deficiency and AAT induce additive anti-angiogenic effects by reducing endothelial cell proliferation without affecting vessel functionality. **a** Histomorphometric quantification of CD31^+^ microvessels in Panc02 tumor sections. Results are shown for C57Bl/6J (WT) and MC-deficient (Wsh) mice treated with placebo or 20 mg/kg DC101 (*n* = 8/8/5/5; *p < 0.05; one-way ANOVA). **b** Representative images of CD31^+^ tumor sections are displayed and their quantification is shown in each panel. *Scale bar* represents 100 µm. **c**–**e** Histomorphometric quantification of BrdU^+^ CD105^+^ proliferating (**c**) and CD31^+^ total (**e**) tumor microvessels (*n* = 7/7/6/6; *p < 0.05; one-way ANOVA). **d** Representative images of BrdU^+^ CD105^+^ tumor sections. *Arrows* indicate examples of proliferating microvessels and their quantification is shown in each panel. Inlets show details of proliferating endothelial cells. *Scale bar* represents 100 µm. Results are shown for C57Bl/6J (WT) and MC-deficient (Wsh) mice transplanted with Panc02 tumors that were treated for 4 or 21 days with placebo or 20 mg/kg DC101. **f**, **g** Histomorphometric quantification of CD31^+^ FITC-Lectin^+^ microvessels in Panc02 tumor sections displayed as absolute (**f**) or relative (**g**) values (*n* = 8/8/5/5; *p < 0.05; one-way ANOVA). **h** Histomorphometric quantification of Pimonidazole^+^ hypoxic areas from Panc02 tumor sections. Results are shown for C57Bl/6J (WT) and MC-deficient (Wsh) mice treated with placebo or 20 mg/kg DC101 (*n* = 6/7/4/4; *p < 0.05; one-way ANOVA). **i** Histomorphometric quantification of BrdU^+^ area from Panc02 tumor sections. Panc02 tumor cells were transplanted alone or in combination with BMMC into the flanks of C57Bl/6J (WT) or MC-deficient (Wsh) mice. After randomization animals received treatment with placebo or 20 mg/kg DC101 (*n* = 9/9/7/6/7/5; *p < 0.05; one-way ANOVA). Results are shown as representative means ± s.e.m

In concordance with literature, MVD was lower in MC-deficient tumors (Fig. 2a, b). Moreover, DC101 treatment was less effective in reducing MVD when MC were present (treated WT: 36 ± 1 vessels/mm^2^, treated Wsh: 21 ± 1 vessels/mm^2^; *n* = 5–8; *p < 0.05; one-way ANOVA). Thus, MC impair DC101-mediated vessel pruning.

Since MC can stimulate the proliferation of endothelial cells (EC) in vitro and in vivo^20, 21^, we investigated whether MC-mediated induction of vessel proliferation counteracts the anti-angiogenic effect of DC101.

Consistent with published data we observed a profound inhibitory effect of DC101 on vessel proliferation after 96 h (Fig. 2c, d) regardless of presence or absence of MC^22^. Interestingly, this anti-proliferative effect of DC101 on EC was completely abrogated after 21 days in WT mice, whereas vessel proliferation further decreased under treatment in the absence of MC. Similar data were obtained upon quantification of MVD (Fig. 2e). These findings suggest that MC might counteract the anti-angiogenic effect of VEGFR2 inhibition by induction of EC proliferation in vivo.

Decreased MVD upon AAT can compromise tumor perfusion, which leads to hypoxia and subsequent therapy resistance^3, 23, 24^. By quantifying FITC-Lectin^+^ perfused vessels in end-stage tumors, we found that DC101 treatment of MC-deficient mice only slightly further reduced the mean number of perfused vessels compared to DC101-treated WT mice (Fig. 2f). Moreover, due to reduced vessel numbers in DC101-treated Wsh mice, the relative fraction of perfused vessels after treatment slightly increased in the absence of MC (Fig. 2g). Consequently, intratumoral hypoxia increased upon treatment of WT mice with DC101 (Fig. 2h); however, hypoxia levels were similar in control-treated Wsh mice and non-significantly increased upon treatment of Wsh mice with DC101. Thus, MC deficiency in combination with DC101 does not further compromise vessel functionality.

Increased tumor angiogenesis promotes proliferation of tumor cells due to enhanced supply of oxygen and nutrients^25^. In concordance, we observed a correlation between MVD and tumor cell proliferation (*r* ^2^ = 0.93; *p < 0.05; Pearson correlation; Supplementary Fig. 2). Consistently, the proliferative fraction of tumors was reduced in Wsh mice compared to WT mice and DC101 treatment decreased tumor cell proliferation further(Fig. 2i). This effect was abrogated after adoptive transfer of MC.

As MC-deficiency impairs tumor growth in vivo, we determined whether the anti-tumor effects of MC-deficiency and DC101 are additive or whether the efficacy of DC101 is higher in the absence of MC. The relative DC101-induced decrease of tumor weight, MVD, vessel proliferation, and tumor proliferation was more pronounced in Wsh than in WT Panc02 tumor-bearing mice (Supplementary Fig. 3a–d). The relative reduction of tumor weight of DC101-treated Panc02 tumors was 49% in MC-deficient mice vs. only 23% in WT mice (*n* = 5; *p < 0.05; one-way ANOVA). Accordingly, in the gain-of-function experiment the efficacy of DC101 vs. placebo control is significantly lower after adoptive transfer of mast cells in comparison to the group without mast cell transfer (Supplementary Fig. 3e). Analyzing the EL4 tumor model revealed an additive effect of DC101 and MC deficiency (Fig. 1d).

Altogether, our data indicate that MC promote vessel proliferation by mechanisms, which act independently of the VEGF–VEGFR2 axis and thereby contribute to decreased sensitivity of tumors towards DC101.

### AAT induces expression of ECM-degrading proteases in mast cells

In a next step we wished to understand how MC mediate vessel proliferation in the presence of DC101. Histomorphometric analyses revealed that AAT does not change number or degranulation status of tumor-infiltrating MC (Supplementary Fig. 4a, b).

**Fig. 3:**
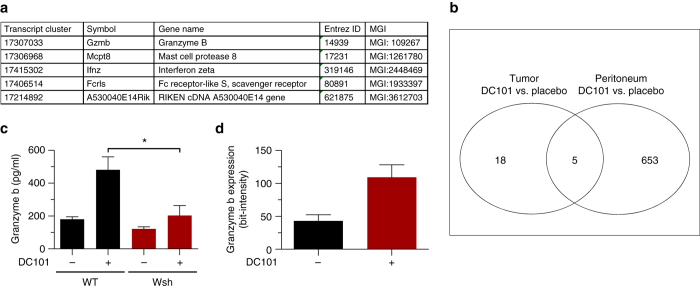
Mast cell-derived granzyme b is upregulated upon AAT. Panc02 cells were transplanted into the flanks of C57Bl/6J mice and animals were treated with placebo or 40 mg/kg DC101. Tumor resident MC were sorted from treated and untreated end-stage tumors as well as from the peritoneal cavity of mice. **a** Differentially regulated genes that overlap in all comparisons. **b** Venn diagram showing the overlap of regulated genes (*p < 0.05; *f*-test). **c** ELISA measuring mouse granzyme b levels in tumor lysates (*n* = 5/5/5/5; *p < 0.05; one-way ANOVA). **d** Histomorphometric quantification of GZMB signal intensities in tryptase^+^ tumor resident MC. Results are shown for C57Bl/6J mice transplanted with Panc02 tumors that were treated with placebo or 20 mg/kg DC101 (*n* = 8/8; *p < 0.05; two-tailed *t*-test). Results are shown as representative means ± s.e.m

Microarray analyses of tumor resident and peritoneal MC indicated that only five genes were significantly deregulated in response to DC101 treatment in both compartments (*p < 0.05, logFC ≥ 1.0; *f*-test) (Fig. 3a, b).

Amongst those genes, mast cell protease 8 (*Mcpt8*) and granzyme b (*Gzmb*) show a high degree of substrate cleavage specificity^26^. Notably, MC-derived GZMB has recently been described to cleave substrates in the extracellular matrix (ECM)^27^. Because this indicates a possible contribution of MC-mediated ECM-remodeling in the development of therapy resistance, we chose to focus on *Mcpt8* and *Gzmb*.

We found that DC101 treatment increased GZMB levels by 2.7-fold (*n* = 5; *p < 0.05; one-way ANOVA) in lysates from tumors grown in WT animals (Fig. 3c) but not in MC-deficient Wsh mice, whereas MCPT8 expression was not modulated (Supplementary Fig. 3c). These data indicate, that increased GZMB levels after DC101 treatment depend on the presence of MC. Quantification of GZMB signal intensities in MC from tumor section confirmed the DC101-induced increase of GZMB in MC (Fig. 3d).

Thus, we hypothesized that GZMB expression by MC might be involved in MC-mediated resistance towards AAT.

### Mast cell-derived GZMB mobilizes ECM-bound FGF-1 and GM-CSF

In order to investigate if GZMB is specifically involved in MC-mediated resistance, we generated *Gzmb* knock down (KD) BMMC by lentiviral transduction (Supplementary Fig. 5).

**Fig. 4:**
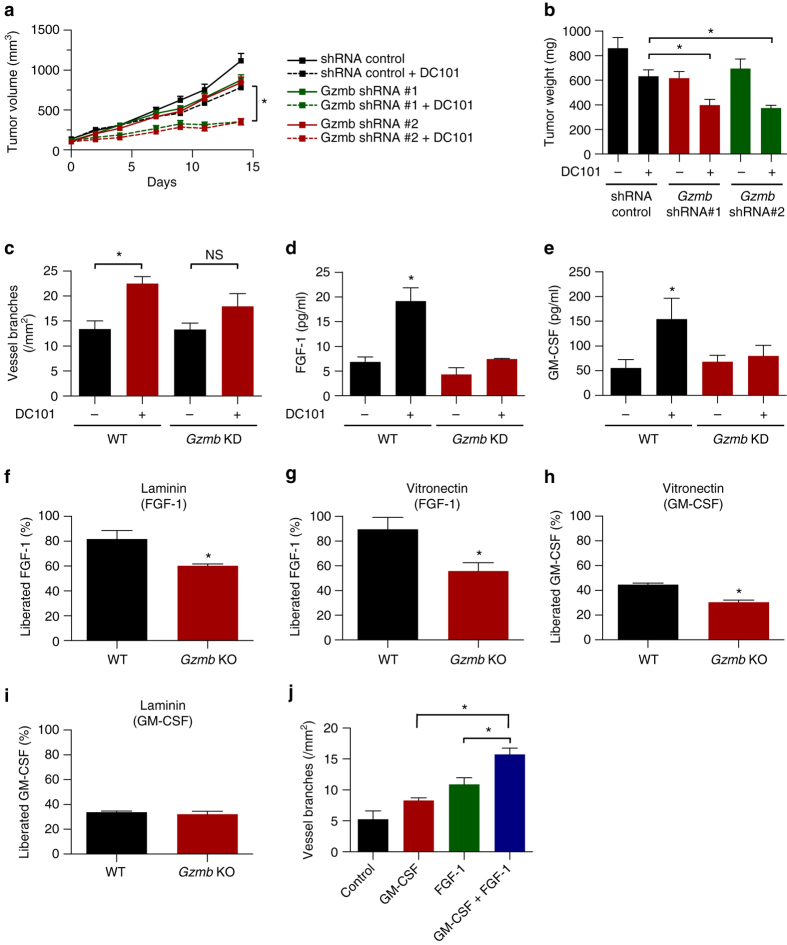
Mast cell-derived granzyme b confers resistance to AAT by releasing ECM-bound FGF-1 and GM-CSF. **a** Subcutaneously growing Panc02 tumors shown as tumor volume. Panc02 cells were injected together with WT- or GZMB KD-BMMC into the flanks of C57Bl/6J (WT) mice. After randomization animals received treatment with placebo or 20 mg/kg DC101 (*n* = 8/8/8/6; *p < 0.05; two-way ANOVA). **b** Weight of end-stage Panc02 tumors at day of euthanasia (*n* = 8/8/5/5; *p < 0.05; one-way ANOVA). **c** Tube formation assays of HUVEC in the presence of tumor-conditioned media from tumors transplanted together with WT and GZMB KD-BMMC and treated with placebo or 20 mg/kg DC101 (*n* = 8/8/8/6; *p < 0.05; one-way ANOVA). **d**, **e** ELISAs showing upregulation of free FGF-1 (**d**) or free GM-CSF (**e**) in tumor-conditioned media (*n* = 3–6; *p < 0.05; one-way ANOVA). **f**–**i** Mobilization of FGF-1 (**f**, **g**) or GM-CSF (**h**, **i**) from a laminin or vitronectin matrix using conditioned media from WT or *Gzmb* KO-BMMC (*n* = 3; *p < 0.05; two-tailed *t*-test. **j** Tube formation assays of HUVEC using 5 ng/ml GM-CSF or FGF-1 (*n* = 3–4; *p < 0.05; one-way ANOVA). Results are shown as representative means ± s.e.m

In contrast to adoptive transfer of WT BMMC, *Gzmb* KD-BMMC failed to decrease efficacy of DC101, suggesting GZMB as a driver of MC-mediated resistance to AAT (Fig. 4a). In line with these data, DC101 decreased tumor weight more pronouncedly when MC were deficient for GZMB (Fig. 4b). Thus MC-derived GZMB decreases sensitivity of tumors towards AAT.

GZMB is able to degrade different substrates in the ECM including laminin and vitronectin which might increase the bioavailability of sequestered pro-angiogenic factors^28, 29^. To approach this question, we generated conditioned media from DC101- and placebo-treated tumors that were co-transplanted with control or *Gzmb* KD-BMMC.

Conditioned media from DC101-treated tumors harboring control BMMC where more potent in inducing tube formation than conditioned media from placebo-treated tumors (Fig. 4c). Interestingly, this increase in pro-angiogenic capacity was not present when tumors received adoptive transfer of *Gzmb* KD-BMMC (Fig. 4c), thereby suggesting GZMB as a mediator of this process.

To identify matrix-bound factors that are liberated by GZMB, we analyzed tumor-conditioned media for pro-angiogenic factors binding to GZMB substrates in the ECM. These assays revealed a DC101-induced increase of free FGF-1 and GM-CSF in the presence of WT BMMC but not *Gzmb* KD-BMMC (Fig. 4d, e). We therefore concluded that MC mobilize FGF-1 and GM-CSF from the ECM in a GZMB-dependent manner.

To pinpoint the ECM component cleaved by GZMB, we focused on extracellular substrates of GZMB with described affinity for different growth factors, laminin and vitronectin^30, 31^. Conditioned medium from WT BMMC was able to release the majority of bound FGF-1 (Fig. 4f) from a laminin matrix into the supernatant (82 ± 7%; *n* = 3; *p < 0.05; two-tailed *t*-test), whereas medium from *Gzmb* KO-BMMC liberated significantly less protein (60 ± 1%; *n* = 3; *p < 0.05; two-tailed *t*-test). Similar data were obtained with vitronectin (Fig. 4g). Moreover, MC-derived GZMB was able to release sequestered GM-CSF from a vitronectin matrix (Fig. 4h), although the effect was not as pronounced as observed with FGF-1. In contrast, GZMB did not contribute to the release of laminin-bound GM-CSF, nor did it mobilize cytokines coated to plastic or the non-GZMB substrate collagen, indicating substrate cleavage and cytokine specificity (Fig. 4i and Supplementary Fig. 6).

As a next step, we analyzed the effect of FGF-1 and GM-CSF on tube formation and found that both mediators induce tube formation in an additive way (Fig. 4j). Thereby they can contribute to GZMB-mediated resistance to AAT.

In summary, we found that tumor-infiltrating MC decrease the sensitivity of tumors towards DC101. MC upregulate GZMB expression, which liberates pro-angiogenic factors such as FGF-1 and GM-CSF from the ECM. Those alternative growth factors can induce vessel proliferation by mechanisms besides the VEGF axis and therefore have the potential to counteract VEGFA–VEGFR2-targeted AAT.

### Mast cells directly degranulate pro-angiogenic factors

We also investigated whether MC can promote resistance towards AAT in a cell autonomous way besides the GZMB-mediated liberation of pro-angiogenic factors. Therefore, we performed matrix-free co-culture experiments with MC generated from murine bone marrow (BMMC) (Supplementary Fig. 1) and human umbilical vein EC (HUVEC).

**Fig. 5:**
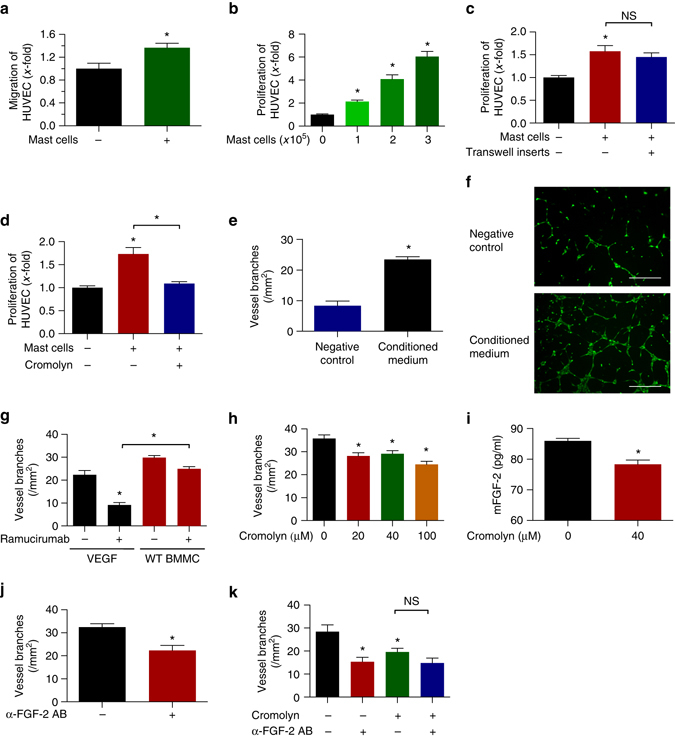
Mast cells secrete pro-angiogenic factors that alter the proliferative and organizational state of endothelial cells. **a** Transwell migration assay of HUVEC stimulated with MC-conditioned medium (*n* = 3; *p < 0.05; two-tailed *t*-test). **b** Direct co-culture of HUVEC and BMMC (*n* = 11–12; *p < 0.05; one-way ANOVA). **c** Indirect co-culture of HUVEC and BMMC (*n* = 3; *p < 0.05; one-way ANOVA). **d** HUVEC were cultured in the presence of MC–conditioned medium (*n* = 3; *p < 0.05; one-way ANOVA). **e** Tube formation assays of HUVEC in the presence of serum free or MC-conditioned medium (*n* = 3–4; *p < 0.05; two-tailed *t*-test). **f** Representative pictures of tube formation assays with *scale bars* representing 400 µm. **g** Tube formation of HUVEC in response to ramucirumab. VEGFA or conditioned medium from WT BMMC was used as stimulus (*n* = 7–10; *p < 0.05; one-way ANOVA). **h** BMMC-induced tube formation of HUVEC response to different concentrations of cromolyn (*n* = 3; *p < 0.05; one-way ANOVA). **i** Secretion of MC-derived FGF-2 in response to 40 µM cromolyn (*n* = 2; *p < 0.05; two-tailed *t*-test). **j** BMMC-induced tube formation of HUVEC in response to 0.5 µg/ml anti-FGF-2 antibody (*n* = 8; *p < 0.05; two-tailed *t*-test). **k** BMMC-induced tube formation of HUVEC in response to single or combinatorial treatment with 20 µM cromolyn and 0.5 µg/ml anti-FGF-2 antibody (*n* = 3; *p < 0.05; one-way ANOVA). Results are shown as representative means ± s.e.m

We observed that MC increase HUVEC migration by 1.4-fold (*n* = 3; *p < 0.05; two-tailed *t*-test) (Fig. 5a). Furthermore, BMMC dose-dependently increase HUVEC proliferation up to 6-fold (*n* = 3; *p < 0.05; one-way ANOVA), a process that was dependent on soluble factors, because cell culture inserts were not able to abrogate it (Fig. 5b, c). In accordance with these data, conditioned media from BMMC could phenocopy this effect (Fig. 5d). Besides EC proliferation, MC–conditioned media led to a 3-fold increase of in vitro angiogenesis in tube-formation assays when compared to non-stimulated EC (Fig. 5e, f).

To answer the question, whether the crosstalk between MC and EC might contribute to resistance towards anti-angiogenic drugs, we performed tube-formation assays using either VEGFA or MC-conditioned medium as a stimulus (Fig. 5g). VEGFR2-targeting with ramucirumab potently abrogated VEGFA-induced but not MC-mediated tube formation, indicating the presence of soluble factors, which rendered MC-mediated tube formation independent of the VEGF(R) axis (Fig. 5g). Importantly, biochemical assays revealed that the presence of MC-derived proteases did not lead to degradation of ramucirumab under conditions similar to those in the in vitro angiogenesis experiments (Supplementary Fig. 7a). To further proof that MC induce VEGFA-independent angiogenesis, we also performed tube formation and cleavage assays with the VEGFA-targeting antibody bevacizumab. These experiments showed similar results as observed with ramucirumab (Supplementary Fig. 7b, c). In addition, we conducted tube-formation assays in the presence of the tyrosine kinase inhibitor (TKI) sunitinib which blocks VEGFR2 besides other TK receptors (Supplementary Fig. 7d). These data showed that sunitinib-treatment had a stronger effect on VEGFA- than on MC-induced tube formation.

In addition, conditioned media from MC that were treated with the degranulation inhibitor cromolyn no longer exerted a pro-proliferative or tube-inducing effect on HUVEC (Fig. 5d, h). This effect was independent of GZMB because GZMB levels in the supernatants of MC cultures were unchanged after treatment with cromolyn (Supplementary Fig. 8a).

We hypothesized that lower amounts of pro-angiogenic mediators might be secreted by MC upon inhibition of degranulation. To analyze this, we investigated the protein levels of pro-angiogenic mediators known to be stored and secreted by MC, such as FGF-2^32^ or HGF^33^. We found that FGF-2 levels were significantly decreased in MC supernatants after treatment with cromolyn while HGF levels were unchanged (Fig. 5i and Supplementary Fig. 8b).

A neutralizing antibody against FGF-2 reduced MC-induced tube formation to a similar extent as observed with cromolyn (Fig. 5j), and the combination of cromolyn and FGF-2 blockade did not induce additive inhibitory effects on tube formation, indicating that FGF-2 release is blocked by cromolyn (Fig. 5k).

Thus, MC also directly secrete pro-angiogenic mediators including FGF-2 in a degranulation-dependent way, thereby decreasing the efficacy of AAT.

### Inhibition of mast cell degranulation increases efficacy of AAT

**Fig. 6:**
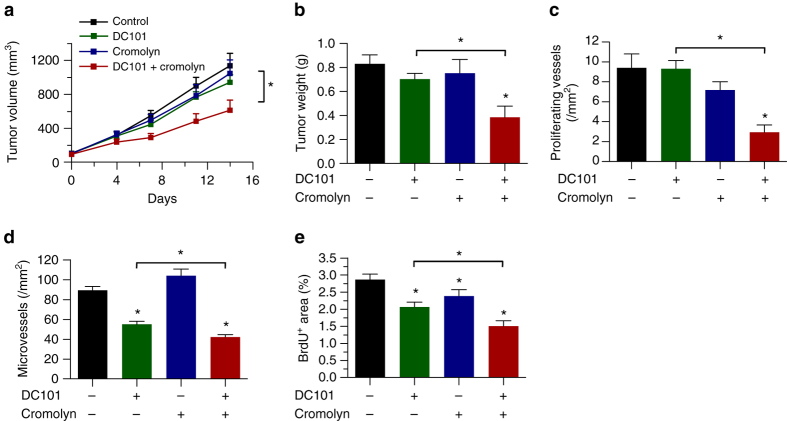
Pharmacological inhibition of mast cell degranulation increases efficacy of AAT. **a** Kinetic of subcutaneously growing Panc02 tumors expressed as tumor volume. Panc02 tumor cells were transplanted into the flanks of C57Bl/6J mice. After randomization, animals were treated with 20 mg/kg DC101 or 25 mg/kg cromolyn in single and combinatorial therapy (*n* = 7/8/7/8; *p < 0.05; two-way ANOVA). **b** Weight of end-stage Panc02 tumors at day of euthanasia (*n* = 8/7/8/7; *p < 0.05; one-way ANOVA). **c**–**e** Histomorphometric quantification of BrdU^+^ CD105^+^ proliferating (**c**) and CD31^+^ total (**d**) tumor microvessels and of BrdU^+^ tumor area (**e**) (*n* = 8/7/8/7; *p < 0.05; one-way ANOVA). Results are shown as representative means ± s.e.m

Next, we investigated whether treatment with cromolyn increases sensitivity of tumors towards AAT in vivo. In concordance with our in vitro data we found an additive inhibitory effect of DC101 and cromolyn on tumor growth in two independent tumor models (Fig. 6a, b; Supplementary Fig. 9).

Histological analyses indicated that DC101 reduced tumor vessel proliferation after long-term treatment only in combination with cromolyn but was ineffective when DC101 was used as monotherapy (Fig. 6c). Furthermore, we detected an additive anti-angiogenic effect that was present when DC101 and cromolyn were combined, which decreased MVD and tumor cell proliferation (Fig. 6d, e). To take into account potential off-target effects of cromolyn, we also determined the efficacy of DC101 alone and in combination therapy by normalizing it to the respective placebo-treated or cromolyn-treated control group. In line with previous data from the genetic model of MC-deficiency, DC101 led to a stronger relative decrease of tumor weight upon pharmacological MC inhibition (Supplementary Fig. 10a). The relative reduction of tumor weight of Panc02 tumors was 49% when combined with cromolyn vs. only 15% in combination with placebo (*n* = 7/8; *p < 0.05; one-way ANOVA). Importantly, this was also true for histological parameters such as microvessel proliferation or MVD (Supplementary Fig. 10b, c). When analyzing tumor cell proliferation an additive effect of the combination therapy was observed (Supplementary Fig. 10d).

Thus, in addition to the increased expression of GZMB upon anti-angiogenic treatment, which liberates pro-angiogenic factors besides the VEGF axis from the ECM, MC also directly decrease sensitivity towards therapy. This effect can be counteracted by the FDA-approved MC stabilizer cromolyn. Therefore, targeting of MC might be a suitable approach to increase efficacy of AAT in the clinic.

## Discussion

The main finding of our study is that MC decrease the efficacy of VEGF(R)-targeted anti-angiogenic therapies by upregulating GZMB, which in turn liberates pro-angiogenic factors besides the VEGF axis from the ECM. In addition, MC directly secrete alternative factors capable of bypassing the VEGF axis. Thereby we uncover a role of MC as mediators of the tumor microenvironment which reduce efficacy of AAT. As pharmacological targeting of MC increased efficacy of AAT, we suggest that this therapeutic combination might be beneficial for cancer patients.

Intensive research efforts have focused on elucidating reasons for the upfront lack or loss of clinical responses to AAT. It was found that cellular components of the tumor microenvironment, an increased cancer stem cell phenotype or the invasive phenotype of cancer cells can mediate resistance against AAT^3, 14, 15^.

We found that MC decrease sensitivity of different experimental tumor models towards AAT because anti-VEGFR2 antibodies (DC101) were more efficiently reducing Panc02 tumor growth in MC-deficient mice compared to WT mice. When analyzing the EL4 tumor model, an additive effect of DC101 and MC deficiency was detectable, although the relative efficacy of DC101 was not significantly increased in the WT mice vs. the Wsh mice. Further work is necessary to determine whether this might be attributed to the rather small DC101-induced anti-tumor effect in this model or if the strength of the MC-mediated response might depend on the tumor type. In any case, the finding that the absence of MC in addition to AAT exerted an additive therapeutic effect is clinically relevant, because MC-targeting would be applied together with AAT.

In in vitro angiogenesis assays DC101 exerted an anti-angiogenic effect against VEGFA but not MC-mediated angiogenesis. Interestingly, tube-formation assays revealed that sunitinib-treatment moderately inhibits MC-induced tube formation, although the effect on VEGFA-induced tube formation was more pronounced. The fact that sunitinib reduces MC-induced tube formation at all can be explained by its off-target activity on other TKI such as PDGFR alpha, PDGFR beta, VEGFR1, FLT3, or c-kit^34^. This implicates that those pathways might also contribute to MC-mediated angiogenesis, which is independent of the VEGFA/VEGFR2-axis.

Many studies have shown that Wsh mice substantially differ from WT mice. The genetic inversion which is responsible for the Wsh-phenotype is accompanied by other pleiotropic effects which go beyond MC-deficiency. As one of the major differentiation marker of the hematopoietic stem and progenitor cell compartment, the lack of c-kit expression has substantial consequences for the hematopoietic system, leading to increased numbers of myeloid cells and megakaryocytes in the spleen of Wsh mice^35^. The same study shows that that the genetic breakpoint of the Wsh mutation disrupts the DNA sequence of the *Corin* gene, thereby leading to cardiac aberrancy^35^. Outside of the hematopoietic system, the impaired c-kit expression in Wsh mice leads to a lack of functional melanocytes^36^. Moreover, c-kit expression has been described for a subpopulation of sensory neurons^37^, keratinocytes^38^, or certain nerves of the central nervous system^39^. Consequently, also other mechanisms besides MC-deficiency could have contributed to the increased efficacy of AAT.

Therefore, it was important to assure that the observed effects are indeed due to the lack of MC. By adoptively transferring in vitro differentiated MC into Wsh mice, we were able to fully rescue the observed phenotype, leading to comparable tumor growth in MC-deficient and WT mice upon treatment with DC101. As the adoptive transfer only reconstituted MC numbers without affecting other pleitropic effects caused by impaired c-kit signaling, it is very likely that MC are the drivers which counteract AAT. However, future studies using c-kit independent models of MC-deficiency are warranted to validate this effect.

We identified two mechanisms how MC reduce efficacy of AAT: (i) the degranulation-independent secretion of GZMB, which liberates alternative pro-angiogenic factors including FGF-1 and GM-CSF from the ECM and (ii) the degranulation-dependent secretion of FGF-2. Via these mechanisms, MC can promote angiogenesis independently of the VEGF(R)-axis and help to bypass therapeutic effects of approved anti-angiogenic drugs all of which target the VEGF pathway.

Interestingly, this enhanced anti-angiogenic effect did not compromise vessel functionality. In fact, the relative decrease of the number of perfused vessels was equally strong in both MC-proficient and MC-deficient mice. We assume that this phenomenon is most likely the reason why DC101 is not more potent in inducing hypoxia in the absence of MC although future studies are needed to unravel the molecular basis for this effect. It is already well known to date that an increase in intratumoral-hypoxia is associated with therapy resistance via different mechanisms^3, 14^. We would therefore anticipate that MC-targeting results in higher efficacy of AAT without increasing hypoxia-elicited resistance mechanisms, which would be desirable in the potential clinical application of this approach.

What are the mechanisms behind GZMB-mediated reduction of therapeutic efficacy? We found that AAT only initially reduced microvessel proliferation thereby reducing MVD. This anti-angiogenic effect was abrogated over time and we provide evidence that MC-derived GZMB is the driver of this process. GZMB expression was recently shown to be present in MC^40^. It was demonstrated that GZMB can liberate VEGF from the ECM^27^; however, this effect is most likely not responsible for the decreased efficacy of DC101 in presence of WT MC because it would be inhibited by our VEGFR2-targeting approach. Searching for other candidates we focused on pro-angiogenic mediators besides the VEGF axis, which can be liberated from known GZMB substrates vitronectin and laminin in the ECM^28, 29^. By analyzing tumor-conditioned media for free pro-angiogenic factors we could detect increased amounts of FGF-1 or GM-CSF in response to DC101 treatment only in the presence of WT- but not GZMB-deficient MC. This GZMB-dependent increase in mobilization of VEGF-independent pro-angiogenic factors could therefore be one reason for reinduction of angiogenesis in the presence of VEGF(R) inhibitors.

In addition, we also found that MC release FGF-2 in a degranulation-dependent manner, because its secretion is impaired upon blockade of degranulation by cromolyn. It was described that cromolyn has significant chelating activity, which can lead to misleading results in vitro. However, for the generation of MC-conditioned media, MC were only pre-treated with cromolyn for 30 min before the cells were centrifuged and resuspended in fresh medium for conditioning. Therefore, only MC but not HUVEC came in contact with cromolyn. Because of this, it is unlikely that the chelating activity of cromolyn interferes with the observed effects on EC.

Our data indicate that FGF-2 is involved in MC-mediated angiogenesis besides the VEGF axis because FGF-2-blocking antibodies counteracted the pro-angiogenic effect of MC-conditioned medium. Blockade of MC degranulation by cromolyn also counteracted the tube-forming activity of MC-conditioned medium, but FGF-2-neutralizing antibodies did not exert an additional inhibition of angiogenesis in this setting. Thus, FGF-2 is secreted from MC in a degranulation-dependent manner. In line with these data, cromolyn and DC101 also exerted additive anti-angiogenic and anti-tumor effects in vivo. This mechanism is independent of GZMB, as inhibition of MC degranulation does not interfere with the release of GZMB.

Of note, recent studies brought up the question about the specificity of cromolyn for MC in mice^41^. To discriminate between additive off-target effects and specific MC-inhibition, we compared the treatment efficacy of DC101 alone and in combination with cromolyn by normalizing it to the respective placebo-treated or cromolyn-treated control group. We found that DC101 is more potent in combination with anti-MC therapy. As these results are in line with the data from the genetic model of MC-deficiency (Wsh), we conclude that the increase of therapeutic efficacy of DC101 is most likely due to the anti-MC effect of cromolyn. However, we cannot entirely rule out a contribution of off-target activity. Therefore, future studies are needed to validate these results using different pharmacological inhibitors of MC-function.

FGF family members have been described to regulate the integrity of existing and the formation of new vessels^42^. For proper function, FGF-1 has to form a complex with heparane sulfate proteoglycans^43, 44^ but also heparin is able to stabilize FGF-1 for dimerization, which is necessary for efficient signaling^45^. MC are a rich source of heparin^46^, suggesting that they can further promote FGF-1 signaling after GZMB-dependent mobilization via induction of protein dimerization. Interestingly, the pro-angiogenic effect of FGF-1 and FGF-2 besides the VEGF–VEGFR2 axis was already demonstrated and combination of the pan-FGFR inhibitor SSR128129E with DC101 in mouse models of pancreatic cancer yielded an additive therapeutic effect^47^.

Also GM-CSF has been described to have a direct effect on EC proliferation and migration in vitro and in vivo^48^ and we could show that the combination of GM-CSF and FGF-1 additively induces tube formation. However, we cannot rule out the possibility that GM-CSF also indirectly influences angiogenesis.

Previous studies showed that also other MC-derived factors such as histamine^49^ or tryptase^50^ are able to promote EC proliferation. Additionally, direct interaction of MC with EC is known to stimulate MC proliferation via the VLA-4/VCAM-1 axis^51^. As a consequence of this vicious circle, increasing numbers of MC secrete higher amounts of pro-angiogenic growth factors capable to foster EC proliferation. In line with this concept, MC density is known to increase during the course of tumor growth^21^. Therefore, the importance of MC-EC crosstalk might also increase over time. It was shown that tumor vessels produce a laminin-rich ECM^52^. Interestingly, laminin is known to be a potent chemo-attractive agent for MC^53^, which implies that EC might recruit MC via secretion of laminin. In line with this theory, tumor MC mostly reside in close proximity to tumor vessels^54^. The tight spatial relation with EC might explain, why a relatively small host cell population such as MC is able to exert a relevant biological effect. The combination of a growth factor-enriched EC matrix with GZMB-producing MC that reside close to vessels allows the tumor vasculature to rapidly adapt to challenging situations without the need of time-consuming and energy consuming protein biosynthesis.

Moreover, by regulating the bioavailability of chemokines, MC have the possibility to further orchestrate the composition of the tumor microenvironment. Besides its pro-angiogenic function, GM-CSF represents a potent chemoattractant for neutrophils, monocytes, and lymphocytes^55^, all of which are known to play distinct roles in tumor angiogenesis^56–58^. Also pro-tumoral MDSC can be recruited into the tumor microenvironment in a MC-dependent manner by secretion of 5-lipoxygenase^59^. Furthermore, MC produce many other proteases besides GZMB, such as tryptases, chymases, or metalloproteases. These enzymes cleave pro-angiogenic factors from extracellular substrates or function as pro-angiogenic mediators themselves^60–62^. However, future studies are warranted to dissect the individual contribution of these enzymes.

With this study, we identified MC as a potential target to increase efficacy of the recently approved monoclonal antibody ramucirumab, which targets VEGFR2. We could demonstrate that a pharmacologic approach using the MC degranulation inhibitor cromolyn had an additive anti-angiogenic and anti-tumor effect when combined with DC101. It is of note, that some FDA-approved tyrosine kinase inhibitors such as ibrutinib^63^ or imatinib^64^ can inhibit the tyrosine kinase c-kit, which is important for MC development and homing into tumors. Ibrutinib in addition inhibits mast cell degranulation, thereby inducing anti-tumor effects in pre-clinical studies^19^. Furthermore, long-term treatment of Ph^+^ chronic myeloid leukemia patients with imatinib led to severe systemic MC deficiency^65^. Targeting MC functionality with ibrutinib, imatinib, or cromolyn could therefore open up therapeutic avenues to sensitize tumor vessels for AAT.

Altogether, MC inhibition holds potential to increase efficacy of AAT. Due to the availability of FDA-approved MC inhibitors our findings could be rapidly translated to the clinics.

## Methods

### Animals

C57BL/6J (WT), Kit^W-sh^/HNihrJaeBsmJ (Wsh) and NOD.Cg-Prkdc^scid^ Il2rg^tm1Wjl^/SzJ (NSG) mice were used. All experiments were carried out in concordance with the institutional guidelines for the welfare of animals in experimental neoplasia and were approved by the local licensing authority (Behörde für Soziales, Gesundheit, Familie, Verbraucherschutz; Amt für Gesundheit und Verbraucherschutz, Hamburg, Germany, project number G036/13 and G126/15).

### Cell culture and in vitro assays

Assays to determine viability, migration, tube formation, or ECM cleavage in single or co-cultures using HUVEC, BMMC, or conditioned media were performed as detailed in the Supplementary Methods. The generation of BMMC was reported previously^66^. MC-specific knock down of *Gzmb* was performed by cloning *Gzmb*-specific shRNAs into retroviral LeGO vectors^67^.

### Conditioned media, ELISA, and qRT-PCR

Conditioned media were generated from BMMC or resected Panc02 tumors. Measurement of GZMB, FGF-1, FGF-2, HGF, and GM-CSF levels were performed using ELISA according to manufacturer’s instructions (R&D Systems, Minneapolis, MN, USA). RNA transcript levels were analyzed with SyBr green qRT-PCR.

### Cancer models and treatments

Human and murine Panc02, EL4, or BxPC3 tumor cell lines were injected alone or in combination with BMMC into WT, Wsh, or NSG mice. The animals were randomized according to tumor volume and standard deviation between the groups and treated with DC101 (20/40 mg/kg), cromolyn (25 mg/kg), or respective placebo control in an unblinded fashion. For histological evaluation animals received 1 mg BrdU (i.p.), 1 mg pimonidazole (i.p.), or 0.05 mg FITC lectin (i.v.) 12 h, 2 h, or 10 min before euthanasia, respectively.

### Immunohistochemistry and morphometric analyses

Tumor sections were stained and analyzed for tumor proliferation (BrdU), vessel number, and vessel proliferation (CD105, R&D Systems, #AF1320 + BrdU, Abd Serotec, #MCA2060), tumor hypoxia (pimonidazole, Hypoxyprobe, #HP3-1000kit), vessel number, and perfusion (CD31, Dianova, #DIA-310 + FITC-lectin, Vector Laboratories, #FL-1171), mast cells (Tryptase, Abcam, #Ab134932), or granzyme b-expressing mast cells (GZMB, R&D Systems, #BAF1865 + Tryptase, Abcam, #Ab134932) as described^68, 69^.

### Microarrays

For gene expression analyses normalized amounts of ST-cDNA were measured with Mouse Gene 2.1 Affymetrix GeneChips. Array image files (CEL files) were processed with R and Bioconductor packages^70^.

### Statistics

Data represent mean ± s.e.m. of representative experiments, unless otherwise stated. To compare the means of two groups, an unpaired, two-tailed student’s *t*-test was used. Pairwise comparison testing in experiments with more than two groups was performed using one-way ANOVA followed by Newman–Keuls post hoc test for multiple comparisons. Pairwise comparisons of tumor growth kinetics were performed using two-way ANOVA followed by Bonferroni post hoc test for multiple comparisons. Statistical significance for Venn-diagrams was calculated using *f*-test. We performed normality testing using D’Agostino and Pearson omnibus normality test wherever possible. Sample sizes were chosen based on previous experience with the used techniques. Samples were excluded from analyses, whenever they were proven to be significant outliers based on Graph Pad 6.0 outlier exclusion software. In all cases statistical significance was assumed when *p* < 0.05. More detailed information can be fou﻿nd in the Supplementary Methods.

### Data availability

Microarray data that support the findings of this study have been deposited in the database NCBI Gene Expression Omnibus with the accession number GSE89395.

## Electronic supplementary material

Supplementary Information
