# Supplementary material for: Mast cells decrease efficacy of anti-angiogenic therapy by secreting matrix-degrading granzyme B
Source: Nat Commun. 2017 Aug 16;8:269. doi: 10.1038/s41467-017-00327-8 (PMC5559596; doi:10.1038/s41467-017-00327-8)
Supplement: Supplementary file 1 — Supplementary Information [file 41467_2017_327_MOESM1_ESM.pdf]

File Name: Supplementary Information
Description: Supplementary Figures, Supplementary Table, Supplementary Methods and Supplementary References

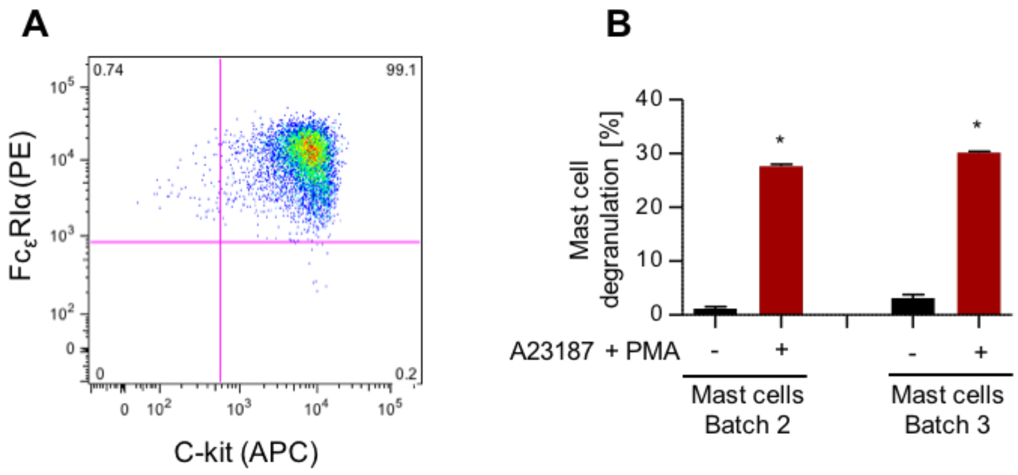

Supplementary Figure 1: *In vitro* differentiation of mast cells from murine bone marrow. (A) Flow cytometric expression analysis of the mast cell marker FcεRIα and c-kit. Double positive cells were considered fully mature mast cells. (B) Biochemical assay that determines ß-hexosaminidase activity in mast cell supernatans. Mast cells were treated with or without 40 µM A23187 and 50 ng/ml PMA to induce degranulation. Degranulation is analyzed by quantification of extra- and intracellular levels of ß-hexosaminidase. Extracellular enzyme levels are displayed as percent of total ß-hexosaminidase (n=3; *p<0.05, two-tailed t-test). Results are shown as representative means ± s.e.m.

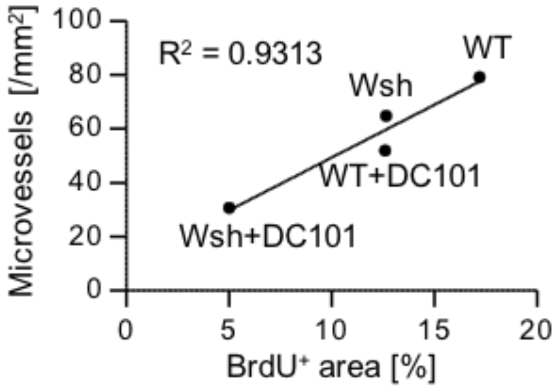

Supplementary Figure 2: Microvessel density correlates with the proliferative fraction of tumors.

Panc02 tumor cells were injected into the flanks of C57Bl/6J (WT) or mast cell deficient (Wsh) mice and animals received treatment with placebo or 20 mg/kg DC101 (n=8/8/5/5; *$p<0.05$; $r^2=0.9313$; Pearson correlation). Histomorphometric quantification of $CD31^+$ microvessels and $BrdU^+$ tumor area was performed and treatment-induced reduction of microvessel density was correlated with the reduction in $BrdU^+$ tumor area. Each dot represents the mean of a treatment group with n≥5 WT or Wsh animals receiving either placebo DC101 (20 mg/kg) treatment. Results are shown as representative means ± s.e.m.

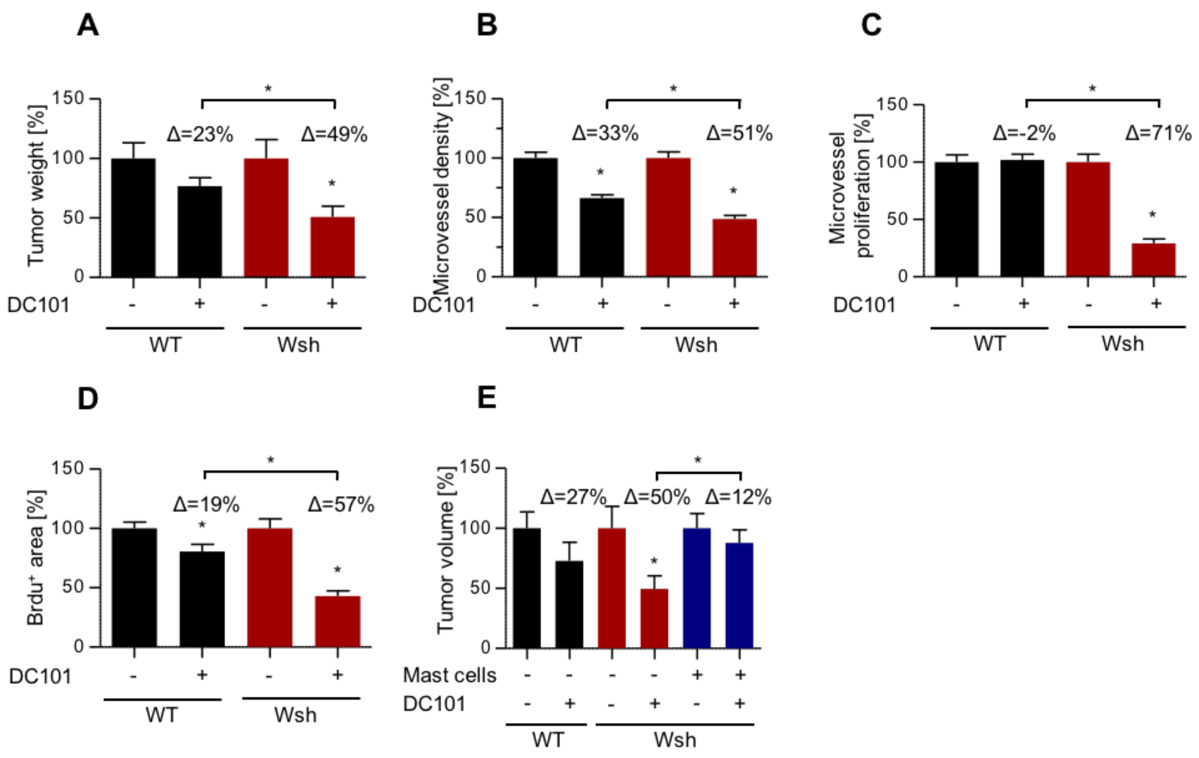

Supplementary Figure 3: Mast cell-deficiency enhances the relative efficacy of AAT.

(A) Relative weight of end stage Panc02 tumors, which were injected into the flanks of C57Bl/6J (WT) or MC deficient (Wsh) mice and received treatment with placebo or 20 mg/kg DC101 (n=5/5/5/5; *p<0.05; two-way ANOVA). (B-D) Ralative histomorphometric quantification of CD31+ total microvessels (B) or CD31+ BrdU+ proliferating microvessels (C) and of BrdU+ proliferating tumor area (D) in Panc02 tumor sections (n=8/8/5/5; *p<0.05; one-way ANOVA). (E) Relative tumor volume of end stage Panc02 tumors with or without adoptive transfer of MC. After randomization animals received treatment with placebo or 20 mg/kg DC101 (n=9/9/7/6/7/5; *p<0.05; one-way ANOVA). Results are shown as representative means ± s.e.m.

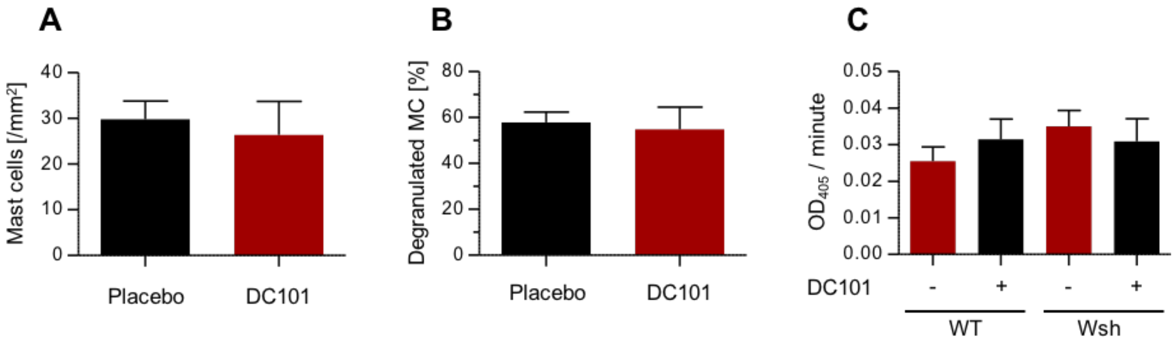

Supplementary Figure 4: Treatment with DC101 does not reduce the number, degranulation status or MCPT8 expression of mast cells.

(A+B) Panc02 tumor cells were injected into the flanks of C57Bl/6J (WT) mice and animals received treatment with placebo or 20 mg/kg DC101 (n=5/5; two-tailed t-test). Mast cell number (A) and degranulation status (B) was determined via histomorhometric analysis of tryptase^{+} cells. (C) Biochemical assay measuring substrate cleavage (Suc-AAPF-ZBzl) by mast cell protease 8 in tumor lysates. Results are depicted as changes in optical density (405 nm) per minute (n=8/8/5/5; one-way ANOVA). Results are shown as representative means ± s.e.m.

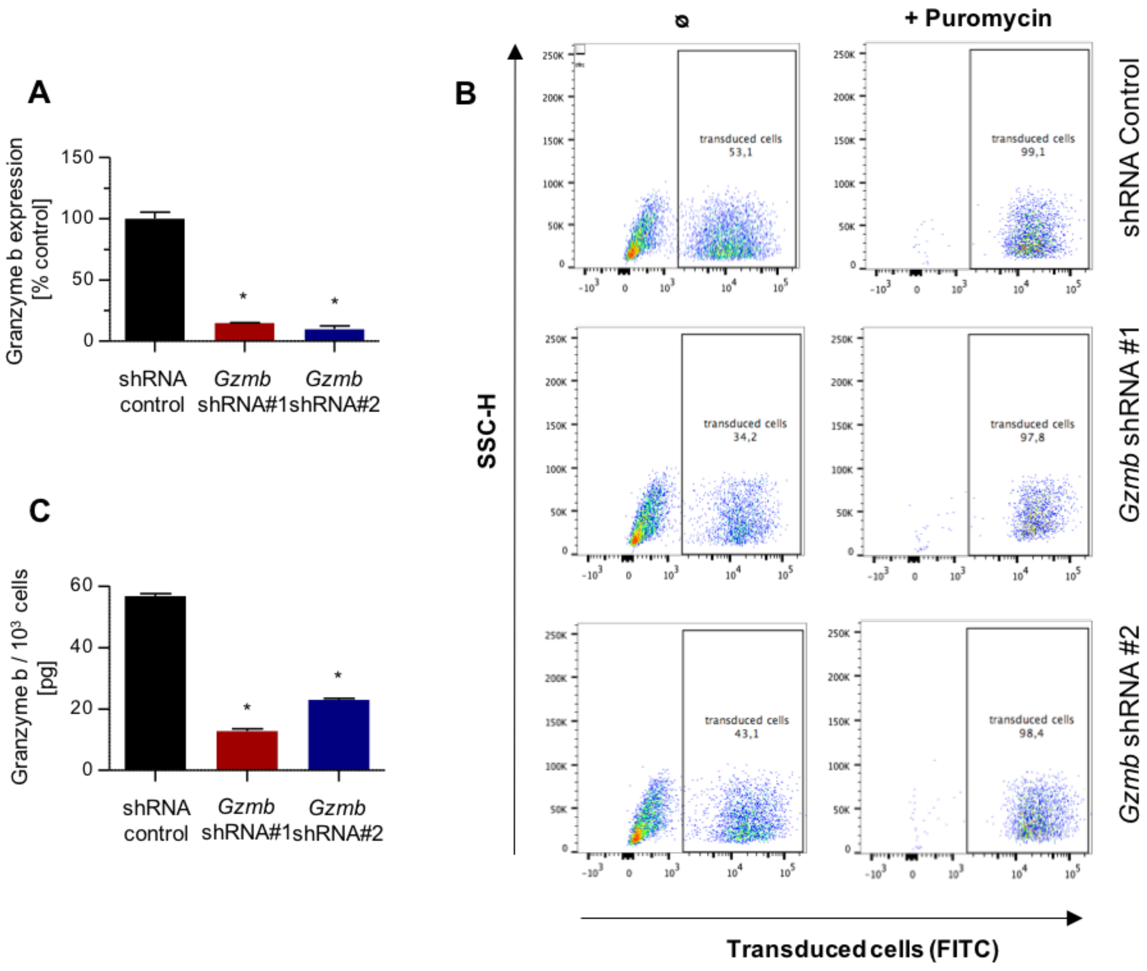

Supplementary Figure 5: Lentiviral knock down of granzyme b in primary bone marrow derived mast cells.

(A) Primary BMMC were transduced with lentiviral particles containing either *Gzmb* shRNA #1-4 or a control construct. Transduced cells were sorted using flow cytometry and qRT-PCR was performed to measure mRNA levels of *Gzmb*. Data were normalized to *Gapdh* as a housekeeping gene and displayed as % of control-transduced cells (n=3; *p<0.05; one-way ANOVA). (B) Transduced primary BMMC that showed the most efficient knock down of *Gzmb* were expanded and selected by adding 5 µg/ml puromycin for 4 days. To determine the purity of cells, cultures were analyzed in a flow cytometer with and without puromycin. (C) Supernatants of mast cell cultures with the most efficient knock down on *Gzmb* were conditioned for 5 days before measuring GZMB levels via ELISA. Data represent the average GZMB-production per 1000 cells (n=3; *p<0.05; one-way ANOVA). Results are shown as representative means ± s.e.m.

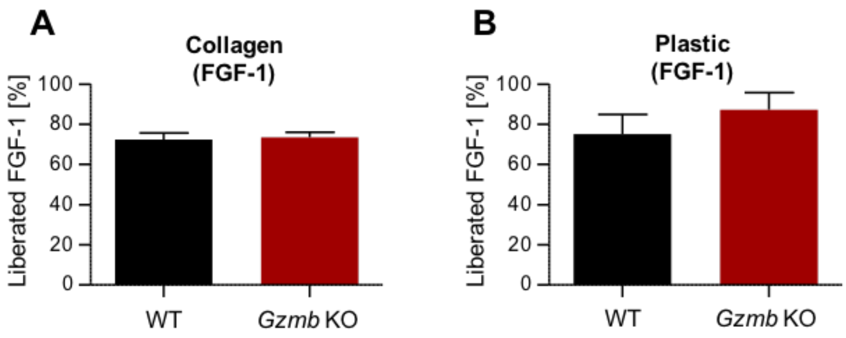

Supplementary Figure 6: Release of FGF-1 by mast cell-derived GZMB is ECM specific. Mobilization of FGF-1 from a collagen matrix (A) or from plastic (B) using conditioned media from WT or *Gzmb* KO BMMC. Determination of protein concentrations was performed by ELISA (n=3; *p<0.05; two-tailed t-test). Results are shown as representative means ± s.e.m.

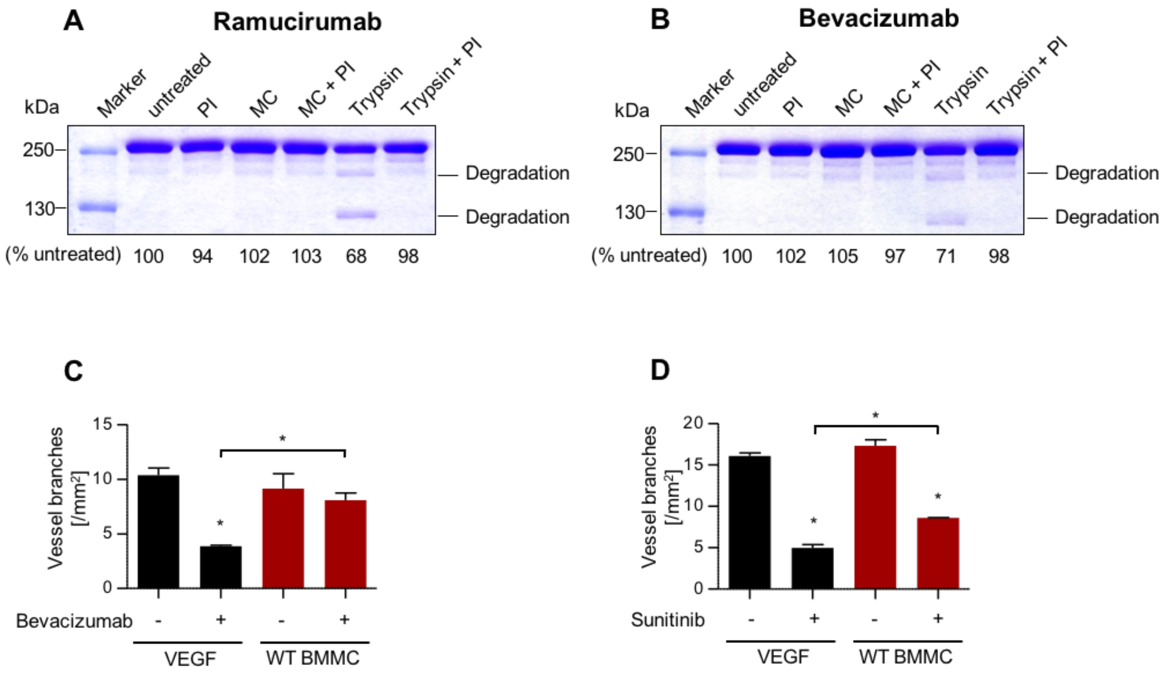

Supplementary Figure 7: Mast cells induce VEGFA-independent tube formation.

(A+B) Immunoprecipitation of ramucirumab (A) or bevacizumab (B) after incubation with MC-conditioned media (MC) or trypsin with and without a pan-protease inhibitor (PI; Complete®, Roche). Full-length antibody was visualized on a coomassie-stained acrylamide gel. (C) Tube formation assays using 200 ng/ml VEGFA or conditioned media from WT bone marrow-derived mast cells (WT BMMC) with and without 2 µg/ml bevacizumab (C) or 25 nM sunitinib (D) (n=3; *$p<0.05$; one-way ANOVA). Results are shown as representative means ± s.e.m.

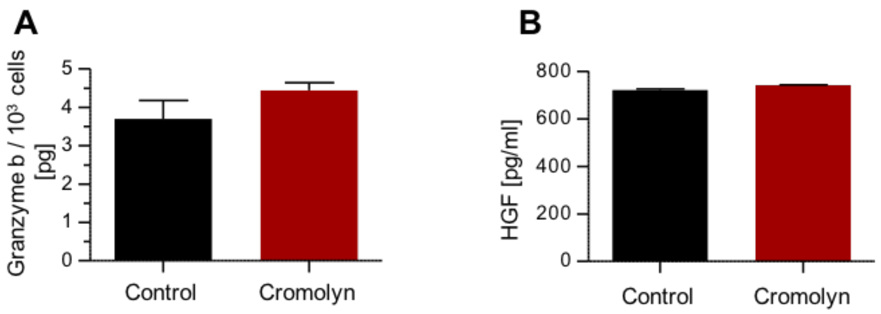

Supplementary Figure 8: Treatment of mast cells with cromolyn does not reduce release of GZMB or HGF.

(A+B) BMMC were treated with 40 µM cromolyn *in vitro*. Supernatants were assayed for the presence of GZMB (A) or HGF (B) using ELISA (n=3; *p<0.05; two-tailed t-test). Results are shown as representative means ± s.e.m.

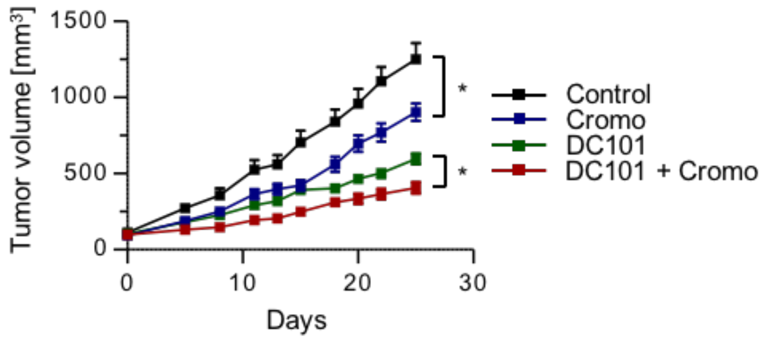

Supplementary Figure 9: Cromolyn treatment sensitizes BxPC3 tumors for anti-angiogenic therapy.

Kinetic of subcutaneously growing BxPC3 tumors expressed as tumor volume. BxPC3 tumor cells were transplanted into the flanks of immunocompromised NSG mice. After randomization animals were treated with 20 mg/kg DC101 or 25 mg/kg cromoglicin in single and combinatorial therapy (n=8/8/6/6; *$p<0.05$). Results are shown as representative means ± s.e.m.

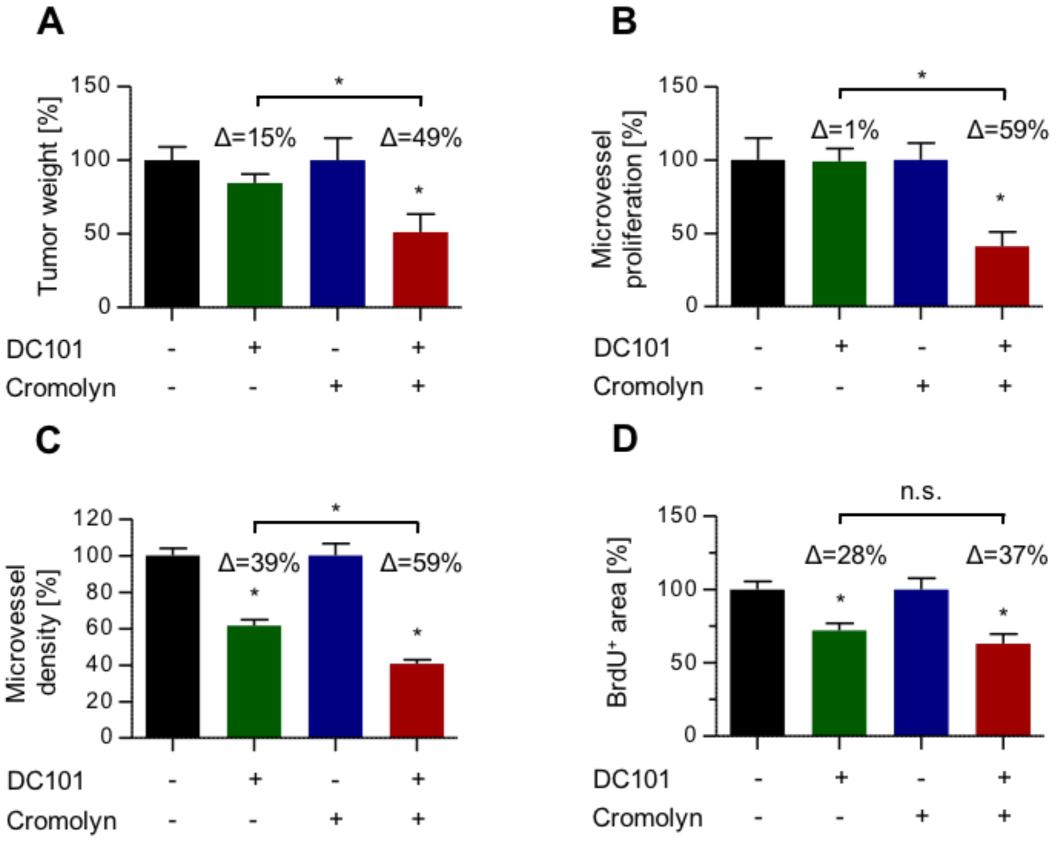

Supplementary Figure 10: Pharmacological inhibition of mast cell-function enhances the relative efficacy of AAT.

Panc02 tumor cells were transplanted into the flanks of C57Bl/6J mice. After randomization, animals were treated with 20 mg/kg DC101 or 25 mg/kg cromolyn in single and combinatorial therapy. (A) Relative weight of end stage Panc02 tumors at day of sacrifice (n=8/7/8/7; *$p<0.05$; one-way ANOVA). (B-D) Relative histomorphometric quantification of BrdU+ CD105+ proliferating (B) or CD31+ total (C) tumor microvessels and of BrdU+ tumor area (D) (n=8/7/8/7; *$p<0.05$; one-way ANOVA). Results are shown as representative means ± s.e.m.

SUPPLEMENTARY METHODS

ANIMALS

Generation of mast cell deficient Kit^{W-sh}/HNihrJaeBsmJ (Wsh) mice was described [1]. The strain was backcrossed into C57BL/6J background for at least 10 generations and obtained from Adrian Liston (VIB, Leuven, Belgium). Immunodeficient NOD.Cg-$Prkdc^{scid}$ $Il2rg^{tm1Wjl}$/SzJ mice (NSG) were obtained from Charles River Laboratories (Sulzfeld, Germany). Breeding of Wsh and NSG mice was carried out in homozygosity in our animal facility (UKE, Hamburg, Germany). C57BL/6 control mice were ordered externally from Charles River Laboratories (Sulzfeld, Germany). Experiments were performed with 7-12 week old female mice.

CELLS AND CULTURE CONDITIONS

Panc02 and EL4 cells were a gift from Prof. Dr. Peter Carmeliet (VIB Vesalius Research Center, KU Leuven). BXPC3 cells were a gift from Prof. Dr. Udo Schumacher (University Medical Center Hamburg-Eppendorf, Hamburg). No cell line used in this study is listed in the ICLAC database of commonly misidentified cell lines. Human BxPC3 cells were authenticated using STR-analysis. All cell lines were routinely tested for contamination with mycoplasma on a regular basis. All cell cultures were maintained at 37°C and 5 % CO_2 in a humidified atmosphere and not cultured longer than 15 passages before experimental use. Murine PancO2 and EL4 cells were cultured in RPMI 1640 medium supplemented with 10 % FCS, 2 mM L-glutamine, 100 U/ml penicillin, 100 µg/ml streptomycin. Human umbilical vein endothelial cells (HUVEC) were cultured in EBM-2 Medium (Lonza Group AG, Basel, Switzerland) supplemented with 2 % FCS, 2 mM L-glutamine, 100 U/ml penicillin, 100 µg/ml streptomycin and a complete set of EGM-2 growth factors (Lonza Group AG, Basel Switzerland). Human BXPC3 cells derived from a patient's pancreatic ductal adenocarcinoma were cultured in RPMI 1640 medium supplemented with 10 % FCS, 2 mM L-glutamine, 100 U/ml penicillin, 100 µg/ml streptomycin. Primary murine bone marrow derived mast cells (BMMC) were generated as described [2] and cultured in DMEM medium supplemented with 10 % FCS, 2 mM L-glutamine,

100 U/ml penicillin, 100 μg/ml streptomycin, 50 μM β-mercaptoethanol, 10 ng/ml murine IL-3, 10 mM HEPES and 1 mM sodium-pyruvate.

LENTIVIRAL KNOCK DOWN OF *GZMB* IN BMMC

To knock down *Gzmb* in BMMC, MISSION shRNA pLKO.1 plasmids harboring two different shRNAs against *Gzmb* were obtained (SHCLND-NM_013542; Sigma-Aldrich, Taufkirchen, Germany). The entire U6 promotor and the shRNA region were amplified from plasmids using the primers p192_GzmB_KD and p263_GzmB_KD (see below) by PCR. The primers introduced restrictions sites for XbaI and SalI, therefore PCR products were cut with XbaI and SalI while the plasmid LeGO-G-Puro$^+$ [3] was cut with XbaI and XhoI. Cut plasmid and PCR product were ligated in a 1:3 molar ratio using T4 ligase (Life technologies, Carlsbad, CA, USA) at 16°C for 90 min.

The resulting ligation product was transformed into One Shot TOP10 Chemically Competent E.coli (Life technologies, Carlsbad, CA, USA). Bacterial clones were picked from ampicillin-containing agar plates and plasmids were purified and analyzed for correct insert via sequencing of cloned regions. VSV-G pseudotyped viral particles were generated as described [4] and used for transduction of primary BMMC according to standard techniques. Transduced BMMC were purified using flow cytometric sorting (DAPI$^-$ eGFP$^+$ FcεRIα$^+$ c-kit$^+$) or selected for 4 days with 5 µg/ml puromycin before experiments were performed. Knock down of *GzmB* was controlled by RT-qPCR. Primers for RT-qPCR are shown in the table below.

Supplementary Table 1: Oligonucleotides for qRT-PCR and cloning.

Name	Purpose	Sequence
**p192_GzmB_KD**	Cloning of gzmB shRNAs	5'-TAC TGC CAT TTG TGT CGA CGT CGA GAA TTC-3'
**p263_GzmB_KD**	Cloning of gzmB shRNAs	5'-GTA TAT CTA GAG GCC GCC CCC TTC ACC GAG-3'
**rt_m_GzmB_fwd**	qRT-PCR	5'-ACA AAG GCA GGG GAG ATC AT-3'
**rt_m_GzmB_rev**	qRT-PCR	5'-CGA ATA AGG AAG CCC CCA CA-3'
**rt_m_Gapdh_fwd**	qRT-PCR	5'-GGT GAA GGT CGG TGT GAA C -3'
**rt_m_Gapdh_rev**	qRT-PCR	5'-GGG GTC TCG CTC CTG GAA -3'

GENERATION OF CONDITIONED MEDIA

For generation of conditioned media for tube formation assays, BMMC were seeded at a density of $2x10^6$ cells/ml in serum free EBM2 medium containing 2 mM L-glutamine, 100 U/ml penicillin, 100 µg/ml streptomycin but no FCS or phenol-red. After treatment with or without 40 µM cromolyn for 30 min, the cells were centrifuged for 5 minutes at 200 x g and re-suspended in serum free EBM2 medium. After conditioning for 72 hours at 37°C and 5 % CO2 in a humidified atmosphere, supernatants were filtered (0.2 µm) and stored at -20°C until use. Before tube formation assays, supernatants were concentrated 5-fold at 1000xg using Amicon Ultra-4 centrifugal filters (Merck KGaA, Darmstadt, Germany) with a molecular cutoff of 10 kDa and stored at -80°C until use.

Generation of conditioned media for proliferation assays was performed by seeding BMMC at a density of $2x10^6$ cells/ml in EBM2 medium containing 2 mM L-glutamine, 100 U/ml penicillin, 100 µg/ml streptomycin and 0.1 % FCS. After treatment with or without 40 µM cromolyn for 30 min, the cells were centrifuged for 5 minutes at 200 x g and re-suspended in EBM2 medium (0.1 % FCS). After conditioning for 12 hours at 37°C and 5 % CO2 in a humidified atmosphere, supernatants were filtered (0.2 µm) and stored at -80°C until use.

For production of tumor-conditioned media, tumors were resected at day of sacrifice, weighed and cut into small pieces. Up to 800 mg of tumor tissue were rinsed in PBS and placed into a well of a 6-well plate. The tissues were covered with 3 ml EBM2 medium per well containing 2 mM L-glutamine, 100 U/ml penicillin, 100 µg/ml streptomycin but no FCS or phenol-red. The plate was incubated for 72 hours at 37°C and 5 % CO2 in a humidified atmosphere. Afterwards, supernatants were taken and centrifuged for 5 minutes at 1000 x g at room temperature. Supernatants were filtered (0.2 µm) and stored at -80°C until use.

CO-CULTURES AND PROLIFERATION ASSAYS

For co-cultivation of HUVEC, cells were starved for 24 h in serum-free medium (0.1 % FCS) and $2x10^4$ HUVEC were seeded in 400 µl serum-free medium into 24-wells and allowed to adhere for 3-5 hours. Medium was removed and $1\text{-}5x10^5$ BMMC or BMMC-conditioned

medium was added resulting in a final volume of 400 µl serum-free medium (0.1 % FCS). Cells were plated in single and co-cultures with or without cell culture inserts and allowed to grow for 48 hours in 37°C and 5 % CO2. Cell proliferation was measured using Wst-1 proliferation reagent (Sigma-Aldrich, Taufkirchen, Germany).

TUBE FORMATION ASSAYS

HUVEC were starved for 24 h in serum free EBM2 medium (0.1 % FCS). Thereafter, Geltrex matrix solution (Thermo Fisher Scientific Inc., Waltham, MA, USA) was thawed on ice and 50 µl Geltrex/48-well were dispensed using precooled pipet tips. The plate was incubated for 30 minutes at 37°C and 5 % CO_2 during which matrix solidification occurred. Cells were detached from culture flask and $2x10^4$ HUVEC were seeded in 100 µl serum-free EBM 2 medium (without FCS, without phenol red) on top of solidified matrices. Afterwards 100 µl of sample were added per well and the plate was incubated overnight at 37°C and 5 % CO_2 with or without 0.5 µg/mL anti-FGF2 antibody (clone bFM-1m, Merck KGaA., Darmstadt, Germany). For analysis of BMMC-mediated resistance to anti-angiogenic therapy, tube formation was induced with 100 µl of BMMC-conditioned medium or 100 ng/ml VEGFA with or without 20 µg/ml ramucirumab. On the next day, the medium was discarded and 200 µl of culture medium with 0.2 µg/ml calcein were added to each well. Samples were stained for 30 minutes at 37°C at 5 % CO_2 before pictures were taken. The pictures were analyzed with ImageJ (National Institute of Health, Washington, DC, USA) by counting vessel branches and nodules per mm^2.

MIGRATION ASSAYS

HUVEC were starved for 12 h in serum free EBM2 medium (0.1 % FCS) and $2x10^4$ cells were seeded into migration inserts (8 µm pore size) of a 24-well plate. Subsequently, $2x10^5$ cells BMMC were seeded below the migration insert and the assays were incubated for 48 h at 37°C. Migration inserts were stained using the Diff Quick kit (Medion diagnostics, Miami, FL, USA) according to manufacturer’s instructions. Non-migrated cells were removed with a cotton

swab before taking pictures. The pictures were analyzed with ImageJ (National Institute of Health, Washington, DC, USA) by counting migrated cells per mm^2.

ECM MOBILIZATION ASSAY

For ECM mobilization assays, the wells of a 24-well plate were coated with laminin (50 µg/ml), vitronectin (1 µg/ml), collagen (50 µg/ml) or 1 % BSA (control) for 24 h. Wells were rinsed three times with PBS and unspecific protein binding was blocked with 500 µl 1 % BSA in PBS for 1 h at RT before matrices were incubated with 300 µl of FGF1 or GM-CSF (25 ng/ml) for 8 h. Spiked matrices were washed three times with PBS and incubated for with 300 µl BMMC-conditioned medium (WT or *Gzmb* KO) or serum free medium over night. On the next day, supernatants were harvested and the remaining ECM was lysed in RIPA buffer. FGF-1 and GM-CSF level in supernatants and matrices were determined by ELISA according to manufacturer's intructions (R&D Systems, Minneapolis, MN, USA).

HISTOLOGY

At the day of sacrifice, tumor samples were fixed overnight in 4 % paraformaldehyde at 4°C prior to paraffin embedding. Paraffin blocks were cut into 4 µm sections and stained for CD31 (clone BAF1865, R&D Systems, Minneapolis, MN, USA), BrdU (clone BU1/75, Bio-Rad AbD Serotec GmbH, Puchheim, Germany), Pimonidazole (clone pAAb2627AP, Hypoxyprobe ltd., Burlington, MA, USA), endoglin (polyclonal, R&D Systems, Minneapolis, MN, USA), granzyme b (polyclonal, R&D Systems, Minneapolis, MN, USA) or tryptase (clone EPR8476, Abcam plc, Cambridge, UK) as described before [5,6].

SUPPLEMENTARY REFERENCES

1 Lyon, M. F. & Glenister, P. H. A new allele sash (Wsh) at the W-locus and a spontaneous recessive lethal in mice. *Genetical research* **39**, 315-322 (1982).

2 Kalesnikoff, J. & Galli, S. J. Antiinflammatory and immunosuppressive functions of mast cells. *Methods in molecular biology* **677**, 207-220, doi:10.1007/978-1-60761-869-0_15 (2011).

3 Weber, K., Mock, U., Petrowitz, B., Bartsch, U. & Fehse, B. Lentiviral gene ontology (LeGO) vectors equipped with novel drug-selectable fluorescent proteins: new building blocks for cell marking and multi-gene analysis. *Gene therapy* **17**, 511-520, doi:10.1038/gt.2009.149 (2010).

4 Weber, K., Bartsch, U., Stocking, C. & Fehse, B. A multicolor panel of novel lentiviral "gene ontology" (LeGO) vectors for functional gene analysis. *Molecular therapy : the journal of the American Society of Gene Therapy* **16**, 698-706, doi:10.1038/mt.2008.6 (2008).

5 Fischer, C. *et al.* Anti-PlGF inhibits growth of VEGF(R)-inhibitor-resistant tumors without affecting healthy vessels. *Cell* **131**, 463-475, doi:10.1016/j.cell.2007.08.038 (2007).

6 Mazzone, M. *et al.* Heterozygous deficiency of PHD2 restores tumor oxygenation and inhibits metastasis via endothelial normalization. *Cell* **136**, 839-851, doi:10.1016/j.cell.2009.01.020 (2009).
